# A Whole-Genome Sequencing Approach To Study Cefoxitin-Resistant Salmonella enterica Serovar Heidelberg Isolates from Various Sources

**DOI:** 10.1128/AAC.01919-16

**Published:** 2017-03-24

**Authors:** Romaine Edirmanasinghe, Rita Finley, E. Jane Parmley, Brent P. Avery, Carolee Carson, Sadjia Bekal, George Golding, Michael R. Mulvey

**Affiliations:** a Department of Medical Microbiology, University of Manitoba, Winnipeg, MB, Canada; b National Microbiology Laboratory, Public Health Agency of Canada, Winnipeg, MB, Canada; c Centre for Food-borne, Environmental and Zoonotic Infectious Diseases, Public Health Agency of Canada, Guelph, ON, Canada; d Institut national de santé publique du Québec, Montréal, QC, Canada

**Keywords:** Québec, Salmonella Heidelberg, beta-lactamase, cefoxitin, plasmid analysis, whole-genome sequencing

## Abstract

This study characterized cefoxitin-resistant and -susceptible Salmonella enterica serovar Heidelberg strains from humans, abattoir poultry, and retail poultry to assess the molecular relationships of isolates from these sources in Québec in 2012. Isolates were collected as part of the Canadian Integrated Program for Antimicrobial Resistance Surveillance (CIPARS). All isolates were subjected to antimicrobial susceptibility testing, PCR for CMY-2, pulsed-field gel electrophoresis (PFGE), and whole-genome sequencing (WGS). A total of 113 *S*. Heidelberg isolates from humans (*n* = 51), abattoir poultry (*n* = 18), and retail poultry (*n* = 44) were studied. All cefoxitin-resistant isolates (*n* = 65) were also resistant to amoxicillin-clavulanic acid, ampicillin, ceftiofur, and ceftriaxone, and all contained the CMY-2 gene. PFGE analysis showed that 111/113 (98.2%) isolates clustered together with ≥90% similarity. Core genome analysis using WGS identified 13 small clusters of isolates with 0 to 4 single nucleotide variations (SNVs), consisting of cefoxitin-resistant and -susceptible human, abattoir poultry, and retail poultry isolates. CMY-2 plasmids from cefoxitin-resistant isolates all belonged to incompatibility group I1. Analysis of IncI1 plasmid sequences revealed high identity (95 to 99%) to a previously described plasmid (pCVM29188_101) found in Salmonella Kentucky. When compared to pCVM29188_101, all sequenced cefoxitin-resistant isolates were found to carry 1 of 10 possible variant plasmids. Transmission of *S*. Heidelberg may be occurring between human, abattoir poultry, and retail poultry sources, and transmission of a common CMY-2 plasmid may be occurring among *S*. Heidelberg strains with variable genetic backgrounds.

## INTRODUCTION

Nontyphoidal Salmonella enterica subsp. enterica is a major cause of human gastroenteritis worldwide (1). In Canada, Salmonella enterica serovar Heidelberg is the third most frequently isolated serovar in humans, behind *S*. Enteritidis and *S*. Typhimurium, which rank as numbers 1 and 2, respectively (2). *S*. Heidelberg is often more invasive than other nontyphoidal serovars and is capable of causing extraintestinal infections and severe septicemia (3). In 2012, 9.24% of *S*. Heidelberg isolates in Canada were recovered from extraintestinal samples in comparison to 3.97% of *S*. Enteriditis and 2.21% of *S*. Typhimurium extraintestinal isolates from the same year (4). In such invasive infections, antimicrobial treatment is important to ensure positive patient outcomes.

Infection with *S*. Heidelberg is linked primarily to the consumption of poultry and is rarely transmitted from person to person (3, 5). Within the Canadian agriculture sector, this serovar is repeatedly isolated from farm, abattoir, and retail poultry samples but less frequently from bovine and porcine samples (6).

Extended-spectrum cephalosporins are used in veterinary medicine for the treatment and prevention of disease in livestock (7). In Canada, ceftiofur, a third-generation cephalosporin, has been employed in an extralabel manner in broiler chickens to prevent omphalitis caused by Escherichia coli. The Canadian Integrated Program for Antimicrobial Resistance Surveillance (CIPARS) (8) observed a strong correlation (*r* = 0.91, *P* < 0.0001) between the prevalence of ceftiofur-resistant *S*. Heidelberg isolates found in retail chicken samples and those found in humans (9). After a voluntary withdrawal of the antimicrobial by provincial hatcheries in 2005, a decrease in ceftiofur-resistant *S*. Heidelberg isolates from chicken meat and humans was observed, which was subsequently followed by an increase in resistant isolates in 2007 that paralleled a partial reinstitution of ceftiofur use (9). Classified by Health Canada as antimicrobials of “high importance” (category 2) (cefoxitin) and “very high importance” (category 1) (ceftriaxone) in human medicine, cefoxitin and ceftriaxone are broad-spectrum second-generation and third-generation cephalosporins, respectively, that are used to treat a wide variety of infections (9, 10).

Resistance of microorganisms to beta-lactam antimicrobials, including cephalosporins, is commonly due to the production of beta-lactamase enzymes that hydrolyze the antimicrobial (11). AmpC beta-lactamases, which belong to Ambler class C enzymes, encode resistance to penicillins, cephalosporins (including cephamycins), and monobactams (12). Although AmpC genes are found intrinsically in some microorganisms, in Salmonella they are due to acquired mechanisms (13). The AmpC beta-lactamase *bla*_CMY-2_, which is commonly found on plasmids, encodes resistance to cefoxitin and is a significant source of beta-lactam resistance in Salmonella found worldwide (14, 15). The *bla*_CMY-2_-containing plasmids can also harbor resistance genes to other classes of antimicrobials, thus potentially conferring multidrug resistance (13).

In this study, we examined the molecular characteristics of *S*. Heidelberg to assess the relationships of isolates from human, abattoir poultry, and retail poultry sources from Québec by using high-quality core genome single-nucleotide variant (hqSNV) analysis and *bla*_CMY-2_ plasmid analysis.

## RESULTS

### Initial characterization of isolates.

There were 128 *S*. Heidelberg isolates tested from human sources, with 31 (24.2%) resistant to cefoxitin, identified in Québec in 2012. A total of 113 isolates (Table 1) were examined in this study, of which 31 of 51 (60.8%) human isolates were cefoxitin resistant. Thirty-six (70.6%) human isolates were identified from stool, 11 (21.6%) from blood, and 1 (2.0%) from urine, and 3 (5.9%) were of unknown origin. All 18 of the selected abattoir poultry isolates were recovered from chicken cecal content, of which 10 (55.6%) were resistant to cefoxitin. Among the 44 retail poultry isolates examined, 24 (54.5%) were cefoxitin resistant. Thirty-one (70.5%) retail isolates were from a sample of fresh meat (24 from chicken, 7 from turkey), while the remaining 13 (29.5%) were from prepackaged, frozen chicken products (e.g., nuggets).

**TABLE 1:** Characteristics of Salmonella Heidelberg identified in this study

Strain ID	Province^*a*^	Yr	Source	Resistance phenotypes^*b*^	Resistance genotypes^*c*^	ST^*d*^	Plasmid(s)^*e*^	Size of *bla*_CMY-2_ plasmid contig(s) (bp)^*f*^	ST of *bla*_CMY-2_ plasmid^*g*^	Group of *bla*_CMY-2_ plasmid^*h*^	No. on minimum spanning tree
12-0315	QC	2012	Human stool	AMC, AMP, FOX, TIO, CRO	*bla*_CMY-2_	15	IncI1, IncX1, ColRNAI	100,217; 1,331	12	C	2
12-0467	QC	2012	Human stool	AMC, AMP, FOX, TIO, CRO	*bla*_CMY-2_	15	IncI1, IncX1	45,026; 27,243; 20,599; 4,881; 1,656	12	A	4
12-0469	QC	2012	Human stool	AMC, AMP, FOX, TIO, CRO	*bla*_CMY-2_	15	IncI1, IncX1, ColpVC	65,621; 24,827; 7,381; 1,656	12	A	5
12-1195	QC	2012	Human stool	AMC, AMP, FOX, TIO, CRO	*bla*_CMY-2_	15	IncI1, IncX1, ColpVC	48,418; 28,410; 23,779; 1,869; 1,759	12		8
12-2458	QC	2012	Human stool	AMC, AMP, FOX, TIO, CRO	*bla*_CMY-2_	15	IncI1, IncX1, ColpVC	100,376; 1,108	12	C	12
12-2460	QC	2012	Human stool	AMC, AMP, FOX, TIO, CRO	*bla*_CMY-2_	15	IncI1, IncX1, ColRNAI	99,125	12	C	13
12-2552	QC	2012	Human stool	AMC, AMP, FOX, TIO, CRO	*bla*_CMY-2_	15	IncI1, IncX1, ColpVC, Col8282	103,169; 1,108	Untypeable	C	14
12-2554	QC	2012	Human stool	AMC, AMP, FOX, TIO, CRO	*bla*_CMY-2_, *bla*_TEM-1B_	15	IncI1, IncX1, Col156	71,837; 19,355	2		15
12-2694	QC	2012	Human stool	AMC, AMP, FOX, TIO, CRO	*bla*_CMY-2_	15	IncI1, IncX1, ColpVC, ColRNAI	65,427; 27,778; 3,694	12	A	16
12-3755	QC	2012	Human stool	AMC, AMP, FOX, TIO, CRO	*bla*_CMY-2_	15	IncI1, IncX1, ColpVC	97,373	12	A	1
12-4179	QC	2012	Human stool	AMC, AMP, FOX, TIO, CRO	*bla*_CMY-2_	15	IncI1, IncX1, ColpVC	99,601	12	C	24
12-4374	QC	2012	Human stool	AMC, AMP, FOX, TIO, CRO	*bla*_CMY-2_	15	IncI1, IncX1, ColpVC, IncFII	96,728	12	A	1
12-4585	QC	2012	Human stool	AMC, AMP, FOX, TIO, CRO	*bla*_CMY-2_	15	IncI1, IncX1, ColpVC, IncFII	96,616	12	A	1
12-5152	QC	2012	Human stool	AMC, AMP, FOX, TIO, CRO	*bla*_CMY-2_	15	IncI1, IncX1, ColpVC	67,970; 27,244; 1,656	12	A	26
12-5444	QC	2012	Human stool	AMC, AMP, FOX, TIO, CRO	*bla*_CMY-2_	15	IncI1, IncX1, ColpVC	95,989; 1,108	12	A	29
12-6245	QC	2012	Human stool	AMC, AMP, FOX, TIO, CRO	*bla*_CMY-2_	15	ColRNAI	Plasmid integrated into chromosome	Plasmid integrated into chromosome	NA^*h*^	33
12-6507	QC	2012	Human stool	AMC, AMP, FOX, TIO, CRO	*bla*_CMY-2_	15	IncI1, ColpVC, ColRNAI	96,095; 1,116	12	A	35
12-7080	QC	2012	Human stool	AMC, AMP, FOX, TIO, CRO	*bla*_CMY-2_	15	IncI1, IncX1, ColpVC	96,725	12	A	1
12-7329	QC	2012	Human stool	AMC, AMP, FOX, TIO, CRO	*bla*_CMY-2_	15	IncI1, IncX1, ColpVC, ColRNAI	99,244	12		38
12-1016	QC	2012	Human stool	AMC, AMP, FOX, TIO, CRO, (CHL)	*bla*_CMY-2_	15	IncI1, IncX1, ColpVC, ColRNAI	96,047; 1,108	12	A	6
12-5643	QC	2012	Human stool	AMC, AMP, FOX, TIO, CRO, NAL	*bla*_CMY-2_	15	IncI1, IncX1	94,220; 4,699; 1,110	12	A	32
12-1667	QC	2012	Human stool	Susceptible	None	15	IncX1, ColpVC	NA	NA	NA	10
12-2695	QC	2012	Human stool	Susceptible	None	15	IncX1, Col8282	NA	NA	NA	17
12-3136	QC	2012	Human stool	Susceptible	None	15	IncX1	NA	NA	NA	17
12-3227	QC	2012	Human stool	Susceptible	None	15	IncX1, ColpVC	NA	NA	NA	18
12-3327	QC	2012	Human stool	Susceptible	None	15	IncX1	NA	NA	NA	17
12-3383	QC	2012	Human stool	Susceptible	None	15	IncX1, ColpVC	NA	NA	NA	20
12-3458	QC	2012	Human stool	Susceptible	None	15	IncX1, ColpVC	NA	NA	NA	20
12-3461	QC	2012	Human stool	Susceptible	None	15	IncX1, ColpVC	NA	NA	NA	20
12-4367	QC	2012	Human stool	Susceptible	None	15	IncX1, ColpVC	NA	NA	NA	25
12-5334	QC	2012	Human stool	Susceptible	None	15	IncFII	NA	NA	NA	27
12-5632	QC	2012	Human stool	Susceptible	None	15	IncX1, ColpVC	NA	NA	NA	31
12-5634	QC	2012	Human stool	Susceptible	None	15	IncX1, ColRNAI	NA	NA	NA	13
12-6510	QC	2012	Human stool	Susceptible	None	15	IncX1, ColpVC, ColRNAI	NA	NA	NA	36
12-7145	QC	2012	Human stool	Susceptible	None	15	IncX1, ColRNAI	NA	NA	NA	13
12-7730	QC	2012	Human stool	Susceptible	None	15	IncX1, ColpVC	NA	NA	NA	39
12-1063	QC	2012	Human blood	AMC, AMP, FOX, TIO, CRO	*bla*_CMY-2_	15	IncI1, IncX1	81,373; 13,154; 4,699; 1,108	12	A	7
12-1847	QC	2012	Human blood	AMC, AMP, FOX, TIO, CRO	*bla*_CMY-2_, *bla*_TEM-1B_	15	IncI1, IncX1	103,791	12	B	11
12-1959	QC	2012	Human blood	AMC, AMP, FOX, TIO, CRO	*bla*_CMY-2_	15	IncI1, IncX1, ColpVC, ColRNAI	96,095; 1,108	12	A	6
12-3918	QC	2012	Human blood	AMC, AMP, FOX, TIO, CRO	*bla*_CMY-2_	15	IncI1, IncX1	69,872; 27,889; 3,871	12	A	23
12-5335	QC	2012	Human blood	AMC, AMP, FOX, TIO, CRO	*bla*_CMY-2_	15	IncI1, IncX1, ColRNAI	99,577	12	C	28
12-7092	QC	2012	Human blood	AMC, AMP, FOX, TIO, CRO	*bla*_CMY-2_	15	IncI1	99,675	12	C	34
12-0466	QC	2012	Human blood	Susceptible	None	15	IncX1, ColpVC	NA	NA	NA	3
12-3330	QC	2012	Human blood	Susceptible	None	15	IncX1, ColpVC	NA	NA	NA	19
12-5542	QC	2012	Human blood	Susceptible	None	15	IncX1, ColpVC	NA	NA	NA	30
12-6342	QC	2012	Human blood	Susceptible	None	15	IncX1, ColRNAI	NA	NA	NA	34
12-7327	QC	2012	Human blood	Susceptible	None	15	ColRNAI	NA	NA	NA	37
13-0067	QC	2012	Human urine	AMC, AMP, FOX, TIO, CRO	*bla*_CMY-2_	15	IncI1, ColpVC, ColRNAI	99,067; 1,108	12	C	40
12-1666	QC	2012	Human (unknown)	AMC, AMP, FOX, TIO, CRO	*bla*_CMY-2_, *bla*_TEM-1B_	15	IncI1, IncX1	101,107	12	A	9
12-3757	QC	2012	Human (unknown)	AMC, AMP, FOX, TIO, CRO	*bla*_CMY-2_	15	IncI1, IncX1, ColpVC	96,280	12	A	21
12-3792	QC	2012	Human (unknown)	AMC, AMP, FOX, TIO, CRO	*bla*_CMY-2_	15	IncI1, IncX1	100,625; 1,219	12	A	22
N13-02936	ON	2011	Animal cecal content (chicken)	AMC, AMP, FOX, TIO, CRO	*bla*_CMY-2_	15	IncI1, IncX1, ColRNAI	99,572	12	C	88
N13-02941	ON	2011	Animal cecal content (chicken)	AMC, AMP, FOX, TIO, CRO	*bla*_CMY-2_	15	IncI1, IncX1	67,413; 27,194; 1,658; 1,215	12	A	89
N13-02943	ON	2011	Animal cecal content (chicken)	AMC, AMP, FOX, TIO, CRO	*bla*_CMY-2_	15	IncI1, IncX1, ColpVC	98,544; 1,214	12	C	90
N13-02944	NB	2012	Animal cecal content (chicken)	AMC, AMP, FOX, TIO, CRO	*bla*_CMY-2_	15	IncI1, IncX1, ColpVC	96,280	12	A	91
N13-02945	ON	2012	Animal cecal content (chicken)	AMC, AMP, FOX, TIO, CRO	*bla*_CMY-2_	15	IncI1, IncX1	96,628	12	A	92
N13-02946	NB	2012	Animal cecal content (chicken)	AMC, AMP, FOX, TIO, CRO	*bla*_CMY-2_	15	IncI1, IncX1	96,123; 1,108	12	A	93
N13-02947	ON	2012	Animal cecal content (chicken)	AMC, AMP, FOX, TIO, CRO	*bla*_CMY-2_	15	IncI1, IncX1, ColRNAI	96,740	12	A	94
N13-02948	ON	2012	Animal cecal content (chicken)	AMC, AMP, FOX, TIO, CRO	*bla*_CMY-2_	15	IncI1, IncX1, ColRNAI	95,999; 1,108	12	A	94
N13-02949	ON	2012	Animal cecal content (chicken)	AMC, AMP, FOX, TIO, CRO	*bla*_CMY-2_	15	IncI1, IncX1, ColpVC	98,943; 1,108	12	C	95
N13-02934	NB	2011	Animal cecal content (chicken)	AMC, AMP, FOX, TIO, CRO, CHL, SUL	*aadA1*, *aadA2*, *bla*_CMY-2_, *cmlA1*, *sul3*	15	IncI1, IncX1, ColpVC	112,670	26		87
N13-01291	QC	2012	Animal cecal content (chicken)	Susceptible	None	15	IncX1, ColpVC	NA	NA	NA	42
N13-01311	QC	2012	Animal cecal content (chicken)	Susceptible	None	15	IncX1	NA	NA	NA	55
N13-01312	QC	2012	Animal cecal content (chicken)	Susceptible	None	15	IncX1, IncX4	NA	NA	NA	56
N13-01323	QC	2012	Animal cecal content (chicken)	Susceptible	None	15	IncX1, ColpVC, ColRNAI	NA	NA	NA	64
N13-01324	QC	2012	Animal cecal content (chicken)	Susceptible	None	15	IncX1, ColpVC, ColRNAI	NA	NA	NA	65
N13-01325	QC	2012	Animal cecal content (chicken)	Susceptible	None	15	IncX1, ColpVC	NA	NA	NA	66
N13-01330	QC	2012	Animal cecal content (chicken)	Susceptible	None	15	IncX1, ColpVC	NA	NA	NA	70
N13-01348	QC	2012	Animal cecal content (chicken)	Susceptible	None	15	IncX1, ColpVC	NA	NA	NA	79
N13-01290	QC	2012	Food aliquot (turkey)	AMC, AMP, FOX, TIO, CRO, STR, SUL, TET	*aadA1*, *bla*_CMY-2_, *bla*_TEM-1B_, *strA*, *strB*, *sul1*, *tetB*	15	IncI1, ColpVC, ColRNAI, Col156, IncHI2, IncHI2A	100,326	12	C	41
N13-01313	QC	2012	Food aliquot (turkey)	AMC, AMP, FOX, TIO, CRO, STR, SUL, TET	*aadA1*, *bla*_CMY-2_, *bla*_TEM-1B_, *strA*, *strB*, *sul1*, *tetB*	15	IncI1, ColpVC, ColRNAI, Col156, IncHI2, IncHI2A	82,151; 12,485; 5,833	25		57
N13-01327	QC	2012	Food aliquot (turkey)	AMC, AMP, FOX, TIO, CRO, TET	*bla*_CMY-2_, *bla*_TEM-1B_, *tetB*	15	IncI1, IncX1	103,902	Untypeable		68
N13-01354	QC	2012	Food aliquot (turkey)	AMC, AMP, FOX, TIO, CRO, STR	*bla*_CMY-2_, *bla*_TEM-1B_, *strA*	15	IncI1, IncX1	103,768	Untypeable	C	84
N13-01326	QC	2012	Food aliquot (turkey)	AMP	*bla*_TEM-1B_	15	IncI1, IncX1, ColRNAI	NA	NA	NA	67
N13-01331	QC	2012	Food aliquot (turkey)	TET	*tetB*	15	IncI1	NA	NA	NA	71
N13-01366	QC	2012	Food aliquot (turkey)	TET	*tetB*	15	IncI1	NA	NA	NA	86
N13-01292	QC	2012	Food aliquot (chicken)	AMC, AMP, FOX, TIO, CRO	*bla*_CMY-2_	15	IncI1, IncX1	100,044; 1,214	12	A	43
N13-01295	QC	2012	Food aliquot (chicken)	AMC, AMP, FOX, TIO, CRO	*bla*_CMY-2_	15	IncI1, IncX1	100,327; 1,210	12	C	28
N13-01297	QC	2012	Food aliquot (chicken)	AMC, AMP, FOX, TIO, CRO	*bla*_CMY-2_	15	IncI1, IncX1	42,796; 27,247; 20,599; 5,280; 3,886	12	A	47
N13-01314	QC	2012	Food aliquot (chicken)	AMC, AMP, FOX, TIO, CRO	*bla*_CMY-2_	15	IncI1, IncX1, ColpVC	96,628; 1,219	12	A	58
N13-01315	QC	2012	Food aliquot (chicken)	AMC, AMP, FOX, TIO, CRO	*bla*_CMY-2_	15	IncI1, IncX1, ColpVC	97,364	12	A	59
N13-01316	QC	2012	Food aliquot (chicken)	AMC, AMP, FOX, TIO, CRO	*bla*_CMY-2_	15	IncI1, IncX1, ColpVC, ColRNAI	96,083; 1,108	12	A	60
N13-01320	QC	2012	Food aliquot (chicken)	AMC, AMP, FOX, TIO, CRO	*bla*_CMY-2_	15	ColRNAI, Col156	Plasmid integrated into chromosome	Plasmid integrated into chromosome	NA	62
N13-01321	QC	2012	Food aliquot (chicken)	AMC, AMP, FOX, TIO, CRO	*bla*_CMY-2_	15	IncI1, IncX1	132,420	66		63
N13-01336	QC	2012	Food aliquot (chicken)	AMC, AMP, FOX, TIO, CRO	*bla*_CMY-2_	15	IncI1, IncX1, ColRNAI	99,636; 1,214	12	C	74
N13-01342	QC	2012	Food aliquot (chicken)	AMC, AMP, FOX, TIO, CRO	*bla*_CMY-2_	15	IncI1, IncX1, ColpVC, Col156	98,725	12	C	77
N13-01352	QC	2012	Food aliquot (chicken)	AMC, AMP, FOX, TIO, CRO	*bla*_CMY-2_	15	IncI1, IncX1, ColRNAI	100,330	12	C	82
N13-01333	QC	2012	Food aliquot (chicken)	AMP	*bla*_TEM-1B_	15	IncI1, IncX1, ColpVC, ColRNAI, Col8282	NA	NA	NA	73
N13-01293	QC	2012	Food aliquot (chicken)	Susceptible	None	15	IncX1, ColpVC	NA	NA	NA	44
N13-01294	QC	2012	Food aliquot (chicken)	Susceptible	None	15	IncX1, ColpVC, ColRNAI	NA	NA	NA	45
N13-01296	QC	2012	Food aliquot (chicken)	Susceptible	None	15	IncX1, ColpVC	NA	NA	NA	46
N13-01298	QC	2012	Food aliquot (chicken)	Susceptible	None	15	IncX1, ColpVC	NA	NA	NA	48
N13-01317	QC	2012	Food aliquot (chicken)	Susceptible	None	15	IncX1, ColpVC	NA	NA	NA	61
N13-01329	QC	2012	Food aliquot (chicken)	Susceptible	None	15	IncX1, ColpVC	NA	NA	NA	69
N13-01332	QC	2012	Food aliquot (chicken)	Susceptible	None	15	None	NA	NA	NA	72
N13-01337	QC	2012	Food aliquot (chicken)	Susceptible	None	15	ColpVC	NA	NA	NA	75
N13-01338	QC	2012	Food aliquot (chicken)	Susceptible	None	15	IncX1, ColpVC	NA	NA	NA	76
N13-01346	QC	2012	Food aliquot (chicken)	Susceptible	None	15	IncX1, ColpVC	NA	NA	NA	78
N13-01351	QC	2012	Food aliquot (chicken)	Susceptible	None	15	None	NA	NA	NA	81
N13-01353	QC	2012	Food aliquot (chicken)	Susceptible	None	15	IncX1, ColpVC, ColRNAI	NA	NA	NA	83
N13-01303	QC	2012	Food prepackaged (chicken)	AMC, AMP, FOX, TIO, CRO	*bla*_CMY-2_	15	IncI1, IncX1, ColpVC	99,670	12	C	50
N13-01304	QC	2012	Food prepackaged (chicken)	AMC, AMP, FOX, TIO, CRO	*bla*_CMY-2_	15	IncI1, IncX1	73,672; 20,599; 5,280; 1,108	12	A	51
N13-01307	QC	2012	Food prepackaged (chicken)	AMC, AMP, FOX, TIO, CRO	*bla*_CMY-2_	15	IncI1, IncX1, ColpVC, ColRNAI	96,083; 1,108	12	A	16
N13-01308	QC	2012	Food prepackaged (chicken)	AMC, AMP, FOX, TIO, CRO	*bla*_CMY-2_	15	IncI1, IncX1, ColpVC, ColRNAI	96,616	12	A	16
N13-01309	QC	2012	Food prepackaged (chicken)	AMC, AMP, FOX, TIO, CRO	*bla*_CMY-2_	15	IncI1, IncX1, ColRNAI	99,236	12	C	54
N13-01318	QC	2012	Food prepackaged (chicken)	AMC, AMP, FOX, TIO, CRO	*bla*_CMY-2_, *bla*_TEM-1B_	15	IncI1, IncX1	103,897	12	B	15
N13-01319	QC	2012	Food prepackaged (chicken)	AMC, AMP, FOX, TIO, CRO	*bla*_CMY-2_, *bla*_TEM-1B_	15	IncI1, IncX1	104,403	12	B	15
N13-01349	QC	2012	Food prepackaged (chicken)	AMC, AMP, FOX, TIO, CRO	*bla*_CMY-2_	15	IncI1, IncX1, ColpVC	97,261	12	A	80
N13-01355	QC	2012	Food prepackaged (chicken)	AMC, AMP, FOX, TIO, CRO	*bla*_CMY-2_	15	IncI1, IncX1	100,219	12	C	85
N13-01301	QC	2012	Food prepackaged (chicken)	Susceptible	None	15	IncX1, ColpVC	NA	NA	NA	49
N13-01305	QC	2012	Food prepackaged (chicken)	Susceptible	None	15	IncX1, ColpVC	NA	NA	NA	52
N13-01306	QC	2012	Food prepackaged (chicken)	Susceptible	None	15	IncX1, ColpVC	NA	NA	NA	53
N13-01322	QC	2012	Food prepackaged (chicken)	Susceptible	None	15	IncX1, ColpVC	NA	NA	NA	20

a NB, New Brunswick; ON, Ontario; QC, Québec.

b AMC, amoxicillin-clavulanic acid; AMP, ampicillin; FOX, cefoxitin; TIO, ceftiofur; CRO, ceftriaxone; CHL, chloramphenicol; NAL, nalidixic acid; STR, streptomycin; SUL, sulfisoxazole; TET, tetracycline. Parentheses indicate intermediate resistance.

c *aadA1-aadA2*, streptomycin adenylytransferase; *bla*_CMY-2_, beta-lactamase; *bla*_TEM-1B_, beta-lactamase; *cmlA1*, chloramphenicol efflux; *strA-strB*, streptomycin phosphotransferase; *sul1-sul3*, dihydropteroate synthase; *tetB*, tetracycline efflux. Determined using the Centre for Genomic Epidemiology's ResFinder program.

d Determined using the Centre for Genomic Epidemiology's MLST program.

e Determined using the Centre for Genomic Epidemiology's PlasmidFinder program.

f Determined using Contiguator.

g Determined using the Centre for Genomic Epidemiology's pMLST program.

h NA, not applicable.

There were 65/113 (57.5%) isolates resistant to amoxicillin-clavulanic acid, ampicillin, cefoxitin, ceftiofur, and ceftriaxone combined. Of these, 6 (9.2%) were also resistant to at least one of the following antimicrobials: nalidixic acid, chloramphenicol, sulfisoxazole, streptomycin, and tetracycline (Table 1). All 65 cefoxitin-resistant isolates were found to harbor *bla*_CMY-2_ using PCR. Nine (13.8%) also tested positive for *bla*_TEM_, and none of the isolates had *bla*_SHV_, *bla*_CTX-M_, or *bla*_OXA-1_.

### Macrorestriction analysis of *S*. Heidelberg.

Macrorestriction analysis using pulsed-field gel electrophoresis (PFGE) of the 113 isolates revealed that all were closely related with ≥80% similarity, with 111 (98.2%) clustering at ≥90% similarity (see Fig. S1 in the supplemental material). Overall there were a total of 16 groups, labeled A1 to A16, where A1 to A4 contained the majority of isolates (*n* = 99, 87.6%). The maximum band difference between A1 and the remaining groups was 6, where most groups (9/16, 56.3%) differed by only 1 to 3 bands.

### WGS analysis of *S*. Heidelberg isolates.

Of the 113 isolates that were analyzed by whole-genome sequencing (WGS), all had an average coverage of 134 times with a range of 56 to 346 times. hqSNV analysis was performed on all isolates, which included *S*. Heidelberg strain 12-4374 as the reference, as this genome was fully closed in a previous study (16). A maximum of 151 hqSNVs were identified between all isolates, with 95.9% of the reference included in the core genome.

**FIG 1:**
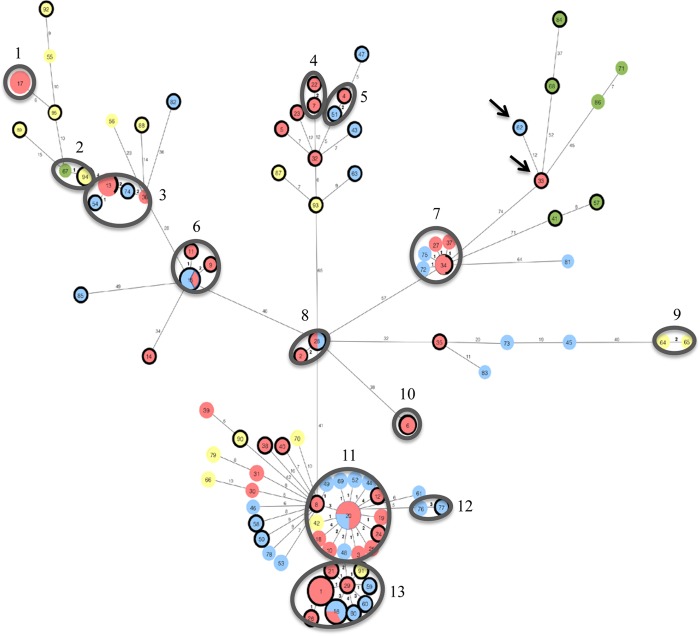
Minimum spanning phylogenetic tree of the core genome of 113 sequenced Salmonella Heidelberg isolates with reference *S*. Heidelberg 12-4374, generated using Phyloviz. Colors for sources of isolates are as follows: red, human; yellow, abattoir chicken; blue, retail chicken; green, retail turkey. Isolates outlined in dark black are resistant to cefoxitin. Isolates in the same circle have 0 hqSNVs, and the size of each circle is proportionate to the number of isolates in the circle. Numbers on branches between 2 isolates represent the number of hqSNV differences. Clusters of isolates outlined in gray indicate those having 0 to 4 hqSNVs and are labeled 1 to 13. The 2 isolates with arrows indicate strains with a portion of a *bla*_CMY-2_ plasmid integrated into the chromosome.

A minimum spanning tree identified 13 clusters containing 2 to 18 isolates (*n* = 68, 60.2%) having between 0 and 4 SNVs (Fig. 1). Two clusters (*n* = 32, 28.3%; clusters 11 and 13) consisted of cefoxitin-resistant and -susceptible human, abattoir chicken, and retail chicken isolates. Cluster 2 contained 2 abattoir chicken isolates clustering with a retail turkey isolate with 1 SNV difference. Among the isolates having 0 SNVs, there were 4 groups (found in clusters 6, 8, 11, and 13) with 2 to 4 isolates each of human and retail chicken origin. Two additional groups having 0 SNVs (found in clusters 3 and 7) with 3 and 2 isolates, respectively, were comprised of cefoxitin-resistant and -susceptible human isolates.

All 113 isolates belonged to multilocus sequence type (MLST) ST15 (Table 1). Predicted antimicrobial resistance genes were identified using WGS data, and the majority of isolates (*n* = 112, 99.1%) had genes that correlated to the antimicrobial phenotypes determined by broth microdilution (Table 1). For the remaining isolate (12-5643), although initial antimicrobial susceptibility testing revealed an increased MIC for nalidixic acid, Resfinder (17) did not detect a corresponding resistance gene for this phenotype. Subsequent retesting using broth microdilution indicated that this isolate was susceptible to nalidixic acid.

### CMY-2 plasmid analysis.

**FIG 2:**
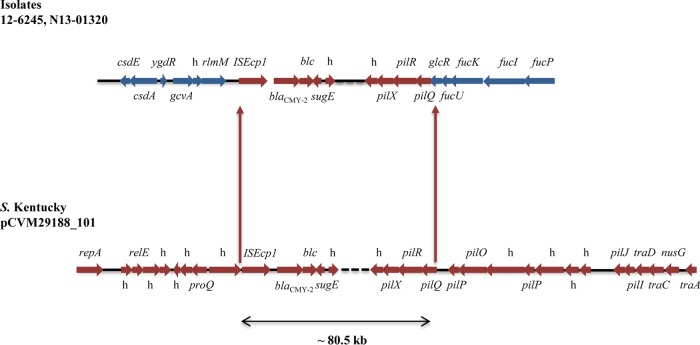
Schematic diagram of plasmid insertion into chromosome for strains 12-6245 and N13-01320 (top). The previously closed IncI1 *bla*_CMY-2_ Salmonella Kentucky pCVM29188_101 plasmid was used as a scaffold to identify the insertions (bottom). Blue arrows represent chromosomal open reading frames (ORFs); red arrows represent plasmid ORFs; h, hypothetical protein.

Using WGS data, 63/65 (96.9%) of the isolates containing *bla*_CMY-2_ plasmids belonged to replicon type IncI1. For the remaining 2 isolates (12-6245 and N13-01320), the *bla*_CMY-2_ gene resided on the largest contig (>747 kb) corresponding to the chromosome, suggesting that these plasmids might have integrated into the chromosome. An alignment of the previously characterized IncI1 *bla*_CMY-2_ plasmid, *S*. Kentucky pCVM29188_101 (GenBank accession number CP001121.1) (18), against the chromosomal contigs identified part of the *bla*_CMY-2_ plasmid (approximately 80.5 kb), which had inserted into the chromosome (Fig. 2). The plasmid inserted 386 bp downstream of the chromosomal *rlmM* gene and into the transcriptional repressor *glcR*, causing the terminal 91 bp to be deleted. No direct or inverted repeats flanking the insertion sites were found. Nucleotide sequence analysis of both 12-6245 and N13-01320 identified IS*Ecp1* approximately 200 bp downstream of the plasmid insertion site, followed by *bla*_CMY-2_ and a portion of the remaining plasmid. Several genes did not integrate with the plasmid into the chromosome, including those belonging to *pil* and *tra* operons, which are associated with transfer pili (Fig. 2). The plasmid replication initiation gene *repA* also did not insert into the chromosome, which may explain the lack of an IncI1 replicon type found in these isolates. The chromosomal insertion site for both isolates was confirmed using PCR. Using the hqSNV analysis, there were 12 SNVs found between isolates 12-6245 and N13-01320 containing the chromosomally located *bla*_CMY-2_ (Fig. 1).

Using plasmid multilocus sequence types (pMLST) based on WGS data, the primary IncI1 plasmid sequence type of the 63 *bla*_CMY-2_ plasmids was ST12 (*n* = 56, 88.9%) (Table 1). Four (6.3%) plasmids were identified as belonging to ST2, ST25, ST26, and ST66, and three (4.8%) were untypeable, as only a portion (289/343 bp) of the *ardA* allele was present. The ST2 plasmid was isolated from a human and was resistant to only the beta-lactam antimicrobials. The plasmid with ST26 was isolated from a chicken and was resistant to beta-lactams, chloramphenicol, and sulfisoxazole. The remaining 2 plasmids with ST25 and ST66 were isolated from retail turkey and retail chicken, respectively, and were resistant to beta-lactams.

**FIG 3:**
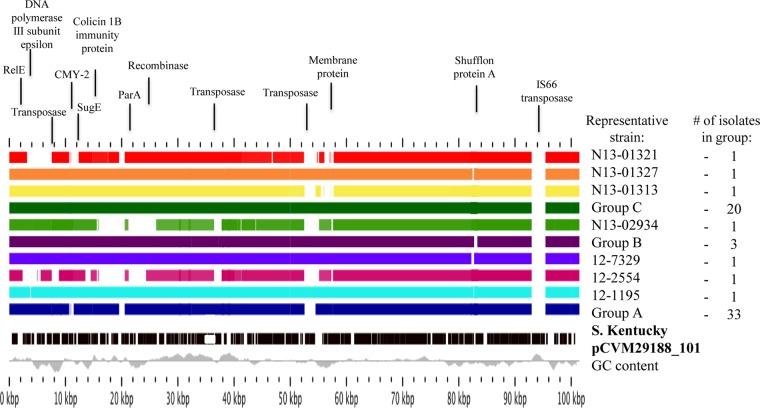
Nucleotide sequence alignments of the previously characterized *S*. Kentucky pCVM29188_101 plasmid against the 10 variant *bla*_CMY-2_-containing plasmids found in all *S*. Heidelberg isolates in this study, generated using GView Server. The reference plasmid is represented by the black boxes that denote open reading frames. For the remaining plasmids, solid colored boxes indicate that DNA is present. The gray line toward the bottom represents the GC content of the reference. Proteins listed above the alignment indicate those that are not present in certain plasmids, except for CMY-2, which was found in all plasmids. A representative sequence of a group A plasmid has been previously published (16).

Sequence analysis of the *bla*_CMY-2_-containing plasmids revealed high homology (95 to 99%) to a previously described plasmid (pCVM29188_101) found in *S*. Kentucky (18). Nucleotide sequence alignments were performed to identify differences in genes compared to pCVM29188_101. All cefoxitin-resistant plasmids in this study belonged to 1 of 10 variant plasmids (Fig. 3). The majority of the 63 plasmids belonged to 1 of 3 variants, labeled as group A (*n* = 33, 52.4%), group B (*n* = 3, 4.8%), and group C (*n* = 20, 31.7%) (Table 1). The 7 remaining plasmids were isolated from individual strains. In comparison to *S*. Kentucky pCVM29188_101, all plasmids were missing the IS*66* transposase. Some plasmids were missing additional genes encoding proteins that included transposases, recombinases, translational repressor protein RelE, DNA polymerase III subunit epsilon, colicin 1B immunity protein, quaternary ammonium resistance protein SugE, chromosome partitioning protein ParA, part of a membrane protein, and part of shufflon protein A.

### Virulence data analysis.

In this study, 169 (8.4%) virulence genes were identified among the 113 strains. These genes were found to be involved in a variety of processes, including adhesion, type III secretion system (T3SS), regulation of genes, resistance to antimicrobial peptides, magnesium uptake, and regulation of stress factors. All sequenced isolates carried genes for curli, fimbriae, Salmonella pathogenicity island 1 and 2, PhoPQ, SodCl, and Mig-14. The majority of isolates (*n* = 97, 85.8%) did not contain *stfA* but contained the other genes in this operon (*stfCDEFG*), which is involved in fimbrial adherence of the organism. Most isolates (*n* = 110, 97.3%) also did not contain *SeAg-B4893*, which encodes a putative outer membrane protein that is involved in fimbrial adherence. Some isolates (*n* = 12, 10.6%) did not carry a greater percentage of virulence genes than the others. The genes missing in these isolates were all different, and they were not associated with any one characteristic, including source, antimicrobial resistance pattern, or type of human infection.

## DISCUSSION

The use of antimicrobials in agriculture is of concern to public health, as overuse or misuse of these drugs can lead to resistance that may transfer to humans (19). A previous study has suggested that the use of ceftiofur in poultry in Québec has contributed to the elevated levels of cephalosporin resistance in *S*. Heidelberg from animals and humans (9). This current study utilized hqSNV analysis of *S*. Heidelberg from humans, abattoir poultry, and retail poultry to further understand the genetic relatedness between isolates from these sources in Québec in 2012.

As stated in previous studies (20), our findings support the fact that macrorestriction analysis using PFGE lacked the discriminatory power that was needed to identify potential relationships of this clonal serovar retrieved from human, abattoir poultry, and retail poultry sources, in contrast to hqSNV analysis using WGS.

Currently, there exists no defined range for the number of SNVs observed between 2 *S*. Heidelberg isolates that are genetically related. A study by Bekal et al. examined 3 epidemiologically defined outbreaks of *S*. Heidelberg in Québec and found a maximum number of 4 SNVs between isolates belonging to the same outbreak (20). Another study by Leekitcharoenphon et al. used hqSNV to characterize an outbreak of *S*. Heidelberg from the United States in 2011 and identified a maximum of 19 SNVs between their outbreak isolates (21). Using a maximum of 4 SNVs, our guideline to consider potential genetic linkages between isolates, we observed 13 clusters of 2 to 18 isolates each containing 0 to 4 SNVs. Four clusters contained up to 4 isolates each of human and retail chicken origin with 0 SNVs in the core genome. Identification of these clusters with 0 to 4 SNVs suggests that isolates within a cluster potentially originated from a common source.

Two groups of isolates with 0 SNVs (found in clusters 3 and 7) had isolates that were susceptible to all antimicrobials tested, and those that contained an IncI1 *bla*_CMY-2_ plasmid conferring resistance to beta-lactams. As extrachromosomal DNA was not included in the core genome used for the analysis, the occurrence of both antimicrobial-resistant and -susceptible isolates with identical core genomes may be indicative of plasmids being gained or lost, as was previously shown with the loss of an IncI1 *bla*_CMY-2_ plasmid found in Salmonella enterica serovar Bredeney within 49 days in an antimicrobial-free environment (22).

The majority of the turkey isolates (6/7, 85.7%) did not cluster with human or chicken isolates. The turkey isolates had different resistance patterns, including being resistant to beta-lactams, streptomycin, sulfisoxazole, and tetracycline (*n* = 2, 28.6%), resistant to only beta-lactams and either tetracycline or streptomycin (*n* = 2, 28.6%), or resistant to only ampicillin or tetracycline (*n* = 3, 42.9%). The fact that no human *S*. Heidelberg isolates clustered with turkey isolates suggested either that the isolates are less virulent in humans or that, possibly more likely, turkey is consumed less frequently than chicken, so humans are exposed to *S*. Heidelberg from turkey less frequently. In Canada, 85.6% of people reported consuming chicken in the previous 7 days compared to 11.8% who consumed turkey; and in Québec specifically, 86.9% of people reported eating chicken in the previous 7 days compared to 7.5% who consumed turkey (23).

All isolates were screened for virulence genes, which were defined as those that aid an organism to colonize a host, replicate, and cause inflammation, tissue damage, and consequently disease (24). There was no correlation seen between type of infection and virulence gene content.

In this study, two *bla*_CMY-2_ plasmids were found to have inserted into the chromosome. It has been suggested that IS*Ecp1* upstream of *bla*_CMY-2_ is involved in mediating such plasmid insertions (25). We identified 12 SNVs between the 2 isolates containing the plasmid integrations. It is unknown whether these integrations occurred separately at 2 different time points or whether they occurred once and the 2 isolates consequently evolved over time.

Comparison of our *bla*_CMY-2_ plasmids against previously characterized plasmids revealed homology to the *S*. Kentucky pCVM29188_101 plasmid and identified 10 plasmid subtypes. Three of the plasmids (groups A, B, and C) were found in multiple isolates of human, abattoir poultry, and retail poultry origins, and all plasmids in these groups belonged to ST12. The finding that the majority of *bla*_CMY-2_-containing plasmids were related suggests the dissemination of a similar resistant plasmid in variable genetic backgrounds of *S*. Heidelberg isolates.

In conclusion, we have compared cefoxitin-resistant and -susceptible *S*. Heidelberg isolates from humans, abattoir poultry, and retail poultry from a specific year and for a specific region in Canada using hqSNV analysis. Our findings suggest that although there is evidence to imply highly related isolates (0 to 4 SNVs) from human and poultry, suggesting a potential common source, it would seem that the majority of cefoxitin-resistant isolates studied occurred due to the dissemination of a plasmid between different *S*. Heidelberg genetic backgrounds. This suggests that the transmission of *bla*_CMY-2_ is due to the horizontal transfer of an IncI1 plasmid rather than clonal dissemination of a particular *S*. Heidelberg strain. Further WGS studies are under way to examine the temporal and spatial distribution of cefoxitin-resistant *S*. Heidelberg in Canada.

## MATERIALS AND METHODS

### Bacterial isolates.

Salmonella Heidelberg isolates were collected as part of CIPARS (8). A convenience sample of 113 isolates from humans (*n* = 51), abattoir poultry (*n* = 18), and retail poultry (*n* = 44) were studied, with the majority (*n* = 103) from Québec from 2012. Since no cefoxitin-resistant abattoir isolates from Québec in 2012 were available, 10 resistant isolates from chicken from Ontario and New Brunswick in 2011 and 2012 were selected for inclusion.

### Initial characterization of isolates.

Antimicrobial susceptibility testing was performed using the broth microdilution Sensititre Automated Microbiology System (Trek Diagnostic Systems Ltd., Oakwood Village, OH, USA) and the CMV3AGNF panel in accordance with Clinical and Laboratory Standards Institute (CLSI) guidelines (26, 27). Multiplex PCR was performed on all cefoxitin-resistant strains (*n* = 65) to identify common extended-spectrum beta-lactamase (ESBL)/AmpC genes (*bla*_SHV_, *bla*_TEM_, *bla*_CTX-M_, *bla*_OXA-1_, and *bla*_CMY-2_), as described previously (28). The genetic relatedness was evaluated using pulsed-field gel electrophoresis (PFGE) according to the Centers for Disease Control and Prevention (CDC) PulseNet protocol (29). DNA fingerprints were analyzed using BioNumerics version 5.1 (Applied Maths, Saint Martens-Latem, Belgium).

### Whole-genome sequencing.

DNA was extracted using the EpiCentre MasterPure Complete DNA and RNA purification kit (Illumina, Madison, WI, USA). Libraries were prepared using the Nextera XT DNA Sample Prep kit (Illumina, Madison, WI, USA). Sequencing was performed using paired-end reads to obtain an average coverage of >60 times for all isolates.

A comparative hqSNV analysis of the core genome was performed (A. Petkau, Core phylogenomics [https://github.com/apetkau/core-phylogenomics]). Horizontally acquired regions of the reference genome (*S*. Heidelberg 12-4374; GenBank accession CP012924.1) (16) containing prophages, genomic islands, and repeats were identified using PHAST (30), IslandViewer (31), and Nucmer v3.1 (32), respectively, and removed from the analysis. Sequencing reads were mapped to *S*. Heidelberg 12-4374 via SMALT v0.7.6 (33), using a k-mer size of 13 and a step size of 6. Variants were detected using FreeBayes v0.9.8 (34), with a minimum mapping quality of 30, a minimum base quality of 30, and a minimum alternate fraction of 0.75. Phylogenetic relatedness and a minimum spanning tree were constructed using Phyloviz (35).

Sequences were assembled into contigs with SPAdes v3.5.0 (36), and genomes were annotated via Prokka v1.1 (37). Assembled contigs were submitted to the Centre for Genomic Epidemiology's multilocus sequence type (MLST) (38), plasmid MLST (pMLST) (39), PlasmidFinder (39), and ResFinder (17) modules to determine whole-genome sequence types (ST), plasmid ST, existing plasmid replicon types, and resistance genes, respectively. Contiguator (40) was used to identify the contigs containing *bla*_CMY-2_ plasmids. Comparison of *bla*_CMY-2_ plasmids was performed against a previously characterized plasmid, *S*. Kentucky pCVM29188_101 (GenBank accession CP001121.1), using GView Server (41). Virulence genes were determined with an in-house workflow using SRST2 v0.1.4.5 (42), which maps Illumina raw reads against chromosomal and plasmid virulence genes found in the Virulence Factor Database for Salmonella (24). Currently, the database contains 2,017 genes associated with virulence in Salmonella (VFDB—current status [http://www.mgc.ac.cn/VFs/status.htm]).

## Supplementary Material

Supplemental material
