# Supplementary material for: A Whole-Genome Sequencing Approach To Study Cefoxitin-Resistant Salmonella enterica Serovar Heidelberg Isolates from Various Sources
Source: Antimicrob Agents Chemother. 2017 Mar 24;61(4):e01919-16. doi: 10.1128/AAC.01919-16 (PMC5365727; doi:10.1128/AAC.01919-16)
Supplement: Supplemental material [file supp_61_4_e01919-16__index.html]

Supplemental material 

# A Whole-Genome Sequencing Approach To Study Cefoxitin-Resistant Salmonella enterica Serovar Heidelberg Isolates from Various Sources

## Supplemental material

- Supplemental file 1 -

  Supplemental Figure S1

  PDF, 152K
