# Supplementary material for: A Whole-Genome Sequencing Approach To Study Cefoxitin-Resistant Salmonella enterica Serovar Heidelberg Isolates from Various Sources
Source: Antimicrob Agents Chemother. 2017 Mar 24;61(4):e01919-16. doi: 10.1128/AAC.01919-16 (PMC5365727; doi:10.1128/AAC.01919-16)
Supplement: Supplemental material [file AAC.01919-16_zac004176050s1.pdf]

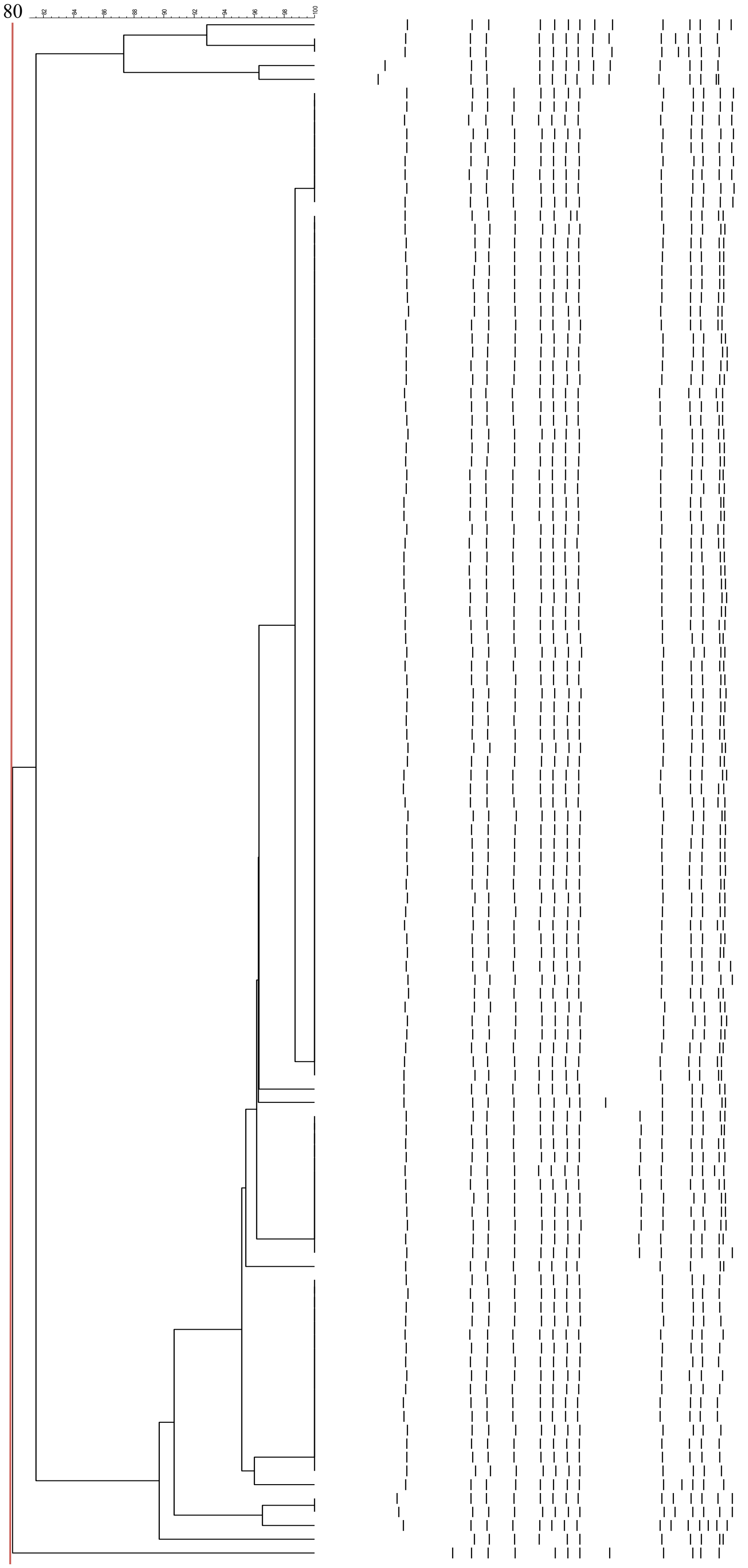

Strain	PFGE group	Source		Province	Resistance phenotype	Year
N13-01327	A5	Food (turkey)	Aliquote	QC	AMC, AMP, FOX, TIO, CRO, TET	2012
N13-01366	A6	Food (turkey)	Aliquote	QC	TET	2012
N13-01331	A6	Food (turkey)	Aliquote	QC	TET	2012
12-6245	A7	Human	Stool	QC	AMC, AMP, FOX, TIO, CRO	2012
N13-01320	A8	Food (chicken)	Aliquote	QC	AMC, AMP, FOX, TIO, CRO	2012
12-0467	A2	Human	Stool	QC	AMC, AMP, FOX, TIO, CRO	2012
12-5643	A2	Human	Stool	QC	AMC, AMP, FOX, TIO, CRO, NAL	2012
12-3792	A2	Human	Unknown	QC	AMC, AMP, FOX, TIO, CRO	2012
N13-01354	A2	Food (turkey)	Aliquote	QC	AMC, AMP, FOX, TIO, CRO, STR	2012
N13-01297	A2	Food (chicken)	Aliquote	QC	AMC, AMP, FOX, TIO, CRO	2012
12-1063	A2	Human	Blood	QC	AMC, AMP, FOX, TIO, CRO	2012
12-3918	A2	Human	Blood	QC	AMC, AMP, FOX, TIO, CRO	2012
N13-01292	A2	Food (chicken)	Aliquote	QC	AMC, AMP, FOX, TIO, CRO	2012
N13-01304	A2	Food (chicken)	Pre-packaged	QC	AMC, AMP, FOX, TIO, CRO	2012
12-3136	A1	Human	Stool	QC	Susceptible	2012
12-3327	A1	Human	Stool	QC	Susceptible	2012
12-3383	A1	Human	Stool	QC	Susceptible	2012
12-3458	A1	Human	Stool	QC	Susceptible	2012
N13-02936	A1	Animal (chicken)	Cecal content	ON	AMC, AMP, FOX, TIO, CRO	2011
N13-02941	A1	Animal (chicken)	Cecal content	ON	AMC, AMP, FOX, TIO, CRO	2011
N13-02944	A1	Animal (chicken)	Cecal content	NB	AMC, AMP, FOX, TIO, CRO	2012
12-1667	A1	Human	Stool	QC	Susceptible	2012
12-2695	A1	Human	Stool	QC	Susceptible	2012
12-2460	A1	Human	Stool	QC	AMC, AMP, FOX, TIO, CRO	2012
12-2552	A1	Human	Stool	QC	AMC, AMP, FOX, TIO, CRO	2012
12-2694	A1	Human	Stool	QC	AMC, AMP, FOX, TIO, CRO	2012
12-4179	A1	Human	Stool	QC	AMC, AMP, FOX, TIO, CRO	2012
12-5152	A1	Human	Stool	QC	AMC, AMP, FOX, TIO, CRO	2012
12-5335	A1	Human	Blood	QC	AMC, AMP, FOX, TIO, CRO	2012
12-7080	A1	Human	Stool	QC	AMC, AMP, FOX, TIO, CRO	2012
12-7329	A1	Human	Stool	QC	AMC, AMP, FOX, TIO, CRO	2012
12-2458	A1	Human	Stool	QC	AMC, AMP, FOX, TIO, CRO	2012
12-3757	A1	Human	Unknown	QC	AMC, AMP, FOX, TIO, CRO	2012
N13-01291	A1	Animal (chicken)	Cecal content	QC	Susceptible	2012
N13-01293	A1	Food (chicken)	Aliquote	QC	Susceptible	2012
N13-01295	A1	Food (chicken)	Aliquote	QC	AMC, AMP, FOX, TIO, CRO	2012
N13-01296	A1	Food (chicken)	Aliquote	QC	Susceptible	2012
N13-01298	A1	Food (chicken)	Aliquote	QC	Susceptible	2012
N13-01301	A1	Food (chicken)	Pre-packaged	QC	Susceptible	2012
N13-01303	A1	Food (chicken)	Pre-packaged	QC	AMC, AMP, FOX, TIO, CRO	2012
N13-01305	A1	Food (chicken)	Pre-packaged	QC	Susceptible	2012
N13-01306	A1	Food (chicken)	Pre-packaged	QC	Susceptible	2012
N13-01307	A1	Food (chicken)	Pre-packaged	QC	AMC, AMP, FOX, TIO, CRO	2012
N13-01308	A1	Food (chicken)	Pre-packaged	QC	AMC, AMP, FOX, TIO, CRO	2012
N13-01311	A1	Animal (chicken)	Cecal content	QC	Susceptible	2012
N13-01337	A1	Food (chicken)	Aliquote	QC	Susceptible	2012
N13-01338	A1	Food (chicken)	Aliquote	QC	Susceptible	2012
N13-01349	A1	Food (chicken)	Pre-packaged	QC	AMC, AMP, FOX, TIO, CRO	2012
N13-01353	A1	Food (chicken)	Aliquote	QC	Susceptible	2012
N13-01355	A1	Food (chicken)	Pre-packaged	QC	AMC, AMP, FOX, TIO, CRO	2012
N13-01323	A1	Animal (chicken)	Cecal content	QC	Susceptible	2012
N13-01325	A1	Animal (chicken)	Cecal content	QC	Susceptible	2012
N13-01324	A1	Animal (chicken)	Cecal content	QC	Susceptible	2012
N13-01326	A1	Food (turkey)	Aliquote	QC	AMP	2012
N13-01329	A1	Food (chicken)	Aliquote	QC	Susceptible	2012
N13-01316	A1	Food (chicken)	Aliquote	QC	AMC, AMP, FOX, TIO, CRO	2012
N13-01317	A1	Food (chicken)	Aliquote	QC	Susceptible	2012
N13-01322	A1	Food (chicken)	Pre-packaged	QC	Susceptible	2012
12-4367	A1	Human	Stool	QC	Susceptible	2012
12-5542	A1	Human	Blood	QC	Susceptible	2012
12-5632	A1	Human	Stool	QC	Susceptible	2012
12-5634	A1	Human	Stool	QC	Susceptible	2012
12-6510	A1	Human	Stool	QC	Susceptible	2012
12-7145	A1	Human	Stool	QC	Susceptible	2012
12-7730	A1	Human	Stool	QC	Susceptible	2012
12-0466	A1	Human	Blood	QC	Susceptible	2012
12-3227	A1	Human	Stool	QC	Susceptible	2012
N13-02947	A1	Animal (chicken)	Cecal content	ON	AMC, AMP, FOX, TIO, CRO	2012
N13-02948	A1	Animal (chicken)	Cecal content	ON	AMC, AMP, FOX, TIO, CRO	2012
12-0469	A2	Human	Stool	QC	AMC, AMP, FOX, TIO, CRO	2012
N13-02934	A2	Animal (chicken)	Cecal content	NB	AMC, AMP, FOX, TIO, CRO, CHL, SUL	2011
N13-02943	A1	Animal (chicken)	Cecal content	ON	AMC, AMP, FOX, TIO, CRO	2011
12-3330	A1	Human	Blood	QC	Susceptible	2012
12-1195	A1	Human	Stool	QC	AMC, AMP, FOX, TIO, CRO	2012
N13-01314	A1	Food (chicken)	Aliquote	QC	AMC, AMP, FOX, TIO, CRO	2012
N13-01348	A1	Animal (chicken)	Cecal content	QC	Susceptible	2012
12-3461	A1	Human	Stool	QC	Susceptible	2012
12-5444	A1	Human	Stool	QC	AMC, AMP, FOX, TIO, CRO	2012
12-0315	A1	Human	Stool	QC	AMC, AMP, FOX, TIO, CRO	2012
N13-01315	A9	Food (chicken)	Aliquote	QC	AMC, AMP, FOX, TIO, CRO	2012
12-1016	A3	Human	Stool	QC	AMC, AMP, FOX, TIO, CRO, (CHL)	2012
12-3755	A3	Human	Stool	QC	AMC, AMP, FOX, TIO, CRO	2012
12-4374	A3	Human	Stool	QC	AMC, AMP, FOX, TIO, CRO	2012
12-4585	A3	Human	Stool	QC	AMC, AMP, FOX, TIO, CRO	2012
12-1959	A3	Human	Blood	QC	AMC, AMP, FOX, TIO, CRO	2012
N13-01309	A3	Food (chicken)	Pre-packaged	QC	AMC, AMP, FOX, TIO, CRO	2012
N13-01312	A3	Animal (chicken)	Cecal content	QC	Susceptible	2012
N13-01346	A3	Food (chicken)	Aliquote	QC	Susceptible	2012
N13-01352	A3	Food (chicken)	Aliquote	QC	AMC, AMP, FOX, TIO, CRO	2012
N13-02949	A3	Animal (chicken)	Cecal content	ON	AMC, AMP, FOX, TIO, CRO	2012
N13-02946	A10	Animal (chicken)	Cecal content	NB	AMC, AMP, FOX, TIO, CRO	2012
N13-01294	A11	Food (chicken)	Aliquote	QC	Susceptible	2012
12-2554	A4	Human	Stool	QC	AMC, AMP, FOX, TIO, CRO	2012
12-7092	A4	Human	Blood	QC	AMC, AMP, FOX, TIO, CRO	2012
12-1666	A4	Human	Unknown	QC	AMC, AMP, FOX, TIO, CRO	2012
N13-01336	A4	Food (chicken)	Aliquote	QC	AMC, AMP, FOX, TIO, CRO	2012
N13-01342	A4	Food (chicken)	Aliquote	QC	AMC, AMP, FOX, TIO, CRO	2012
12-1847	A4	Human	Blood	QC	AMC, AMP, FOX, TIO, CRO	2012
12-6507	A4	Human	Stool	QC	AMC, AMP, FOX, TIO, CRO	2012
N13-01330	A4	Animal (chicken)	Cecal content	QC	Susceptible	2012
N13-01332	A4	Food (chicken)	Aliquote	QC	Susceptible	2012
N13-01318	A4	Food (chicken)	Pre-packaged	QC	AMC, AMP, FOX, TIO, CRO	2012
N13-01319	A4	Food (chicken)	Pre-packaged	QC	AMC, AMP, FOX, TIO, CRO	2012
12-5334	A4	Human	Stool	QC	Susceptible	2012
12-6342	A4	Human	Blood	QC	Susceptible	2012
12-7327	A4	Human	Blood	QC	Susceptible	2012
13-0067	A4	Human	Urine	QC	AMC, AMP, FOX, TIO, CRO	2012
N13-01333	A12	Food (chicken)	Aliquote	QC	AMP	2012
N13-01290	A13	Food (turkey)	Aliquote	QC	AMC, AMP, FOX, TIO, CRO, STR, SUL, TET	2012
N13-01313	A13	Food (turkey)	Aliquote	QC	AMC, AMP, FOX, TIO, CRO, STR, SUL, TET	2012
N13-01321	A14	Food (chicken)	Aliquote	QC	AMC, AMP, FOX, TIO, CRO	2012
N13-02945	A15	Animal (chicken)	Cecal content	ON	AMC, AMP, FOX, TIO, CRO	2012
N13-01351	A16	Food (chicken)	Aliquote	QC	Susceptible	2012

Figure 1. Pulsed-field gel electrophoresis band patterns and dendrogram of 113 *Xba*I digested *Salmonella* Heidelberg isolates. Gels were analyzed using BioNumerics v.5.1, with a band tolerance of 1.5% and optimized at 1.5%, and cluster analysis performed using the Dice coefficient and unweighted pair group method with arithmetic mean (UPGMA). The solid vertical red line indicates isolates having ≥ 80% similarity. Abbreviations of provinces are as follows: NB, New Brunswick; ON, Ontario; QC, Quebec. Abbreviations of antimicrobials are as follows: AMC, amoxicillin/clavulanic acid; AMP, ampicillin; CHL, chloramphenicol; CRO, ceftriaxone; FOX, cefoxitin; NAL, nalidixic acid; STR, streptomycin; SUL, sulfamethoxazole; TET, tetracycline; TIO, ceftiofur.
